# 

**Published:** 1890-01

**Authors:** 


### CONTENTS:

Article I.—Persistent Headaches, and how to Cure them. By Julian J. Chisolm, M. D. - - - 385

" II.—Georgia State Dental Society. Reported by Mrs. M. W. J. - - - - - - - 398

" III.—Antisepsis in General Practice. By Thos. J. Charlton, M. D. - - - - - 414

EDITORIAL.

The Hour at which Death most Usually Occurs. - - - 427

MONTHLY SUMMARY.

Congenital Syphilis in Infants. - - - - - 431

Source of Colors. - - - - - - - - 432

